# Prevalence of *Listeria monocytogenes, Yersinia enterocolitica, Staphylococcus* *aureus*, and *Salmonella* enterica Typhimurium in meat and meat products using multiplex polymerase chain reaction

**DOI:** 10.14202/vetworld.2017.927-931

**Published:** 2017-08-16

**Authors:** C. Latha, C. J. Anu, V. J. Ajaykumar, B. Sunil

**Affiliations:** Department of Veterinary Public Health, College of Veterinary and Animal Sciences, Mannuthy, Thrissur - 680 651, Kerala, India

**Keywords:** food borne pathogens, multiplex polymerase chain reaction, prevalence

## Abstract

**Aim:**

The objective of the study was to investigate the occurrence of *Listeria monocytogenes, Yersinia enterocolitica, Staphylococcus* *aureus*, and *Salmonella enterica* Typhimurium in meat and meat products using the multiplex polymerase chain reaction (PCR) method.

**Materials and Methods:**

The assay combined an enrichment step in tryptic soy broth with yeast extract formulated for the simultaneous growth of target pathogens, DNA isolation and multiplex PCR. A total of 1134 samples including beef (n=349), chicken (n=325), pork (n=310), chevon (n=50), and meat products (n=100) were collected from different parts of Kerala, India. All the samples were subjected to multiplex PCR analysis and culture-based detection for the four pathogens in parallel.

**Results:**

Overall occurrence of *L. monocytogenes* was 0.08 % by cultural method. However, no *L. monocytogenes* was obtained by multiplex PCR method. *Yersinia enterocolitica* was obtained from beef and pork samples. A high prevalence of *S. aureus* (46.7%) was found in all types of meat samples tested. None of the samples was positive for S. Typhimurium.

**Conclusion:**

Multiplex PCR assay used in this study can detect more than one pathogen simultaneously by amplifying more than one target gene in a single reaction, which can save time and labor cost.

## Introduction

The fast and accurate identification of microbial pathogens from food samples by Public Health agencies and diagnostic laboratories ensures not only a better quality of products but also the possibility to adopt timely precautionary measures to limit the spread of infection in case of an outbreak [1]. The introduction of Hazard analysis and critical control points (HACCP) regulations, assigning food safety responsibility to food business operators, underlined that by ensuring the safety and quality of raw materials and the process, the end product can be monitored.

Infections and intoxications caused by foodborne pathogens represent an increasing public health problem. Foods of animal origin, such as meat and meat products, have been reported as the major carriers of these pathogens. Bacterial contamination during slaughtering and processing is a major public health concern and affects shelf life of meat [2]. Use of meat products has increased due to changing food habits. Undercooking and improper storage conditions make meat products more vulnerable to bacterial contamination [3].

Common pathogenic bacteria that cause foodborne diseases include *Listeria monocytogenes, Yersinia enterocolitica, Staphylococcus aureus*, and *Salmonella* spp. *L. monocytogenes* is a major foodborne pathogen which is responsible for high hospitalization rates [4]. Yersiniosis is the third most common zoonoses, and it is most commonly found positive in pig meat and its products [5]. *S. aureus* is often present in food samples and common agents of food poisoning due to the presence of enterotoxins [6,7]. Contamination with staphylococci can be occurred directly from infected food producing animal or at any stages of processing, storage or retailing of food products [8]. *Salmonella* spp. was most frequently detected in poultry meat and less often in pig or bovine meat and also detected at low levels in ready to eat products [5].

The conventional microbiological methods for detection of these bacteria, however, usually include multiple subcultures and biotype or serotype identification steps and, thus are laborious and time-consuming [9]. With the development of molecular techniques, polymerase chain reaction (PCR) has become an important tool for detecting pathogenic microorganisms in food products which improved the sensitivity, specificity, and speed of detection [10]. PCR in multiplex format is needed for the rapid identification of food contaminations caused by more than one microbial species [11].

In this context, the objective of the study was to investigate the occurrence of *L. monocytogenes, Y. enterocolitica, S. aureus*, and *S. enterica* Typhimurium in meat and meat products using the multiplex PCR method. The assay combines an enrichment step in tryptic soy broth with yeast extract (TSBYE) formulated for the simultaneous growth of target pathogens, DNA isolation and multiplex PCR method.

## Materials and Methods

### Ethical approval

Ethical approval was not necessary for this study. However, samples were collected as per standard collection procedure.

### Bacterial strains

The reference strains of bacterial pathogens were procured from MTCC (Microbial type culture collection and Gene bank), Institute of Microbial Technology, Chandigarh India. The reference strains of bacterial pathogens were *L. monocytogenes* (MTCC 1143)*, Y. enterocolitica* (MTCC 859)*, S. aureus* (MTCC 1144) and *S. enterica* Typhimurium (MTCC 98). Pure cultures were maintained in glycerol stocks at −20°C and viability was checked regularly in nutrient agar slants.

### Sample collection

A total of 1134 samples including beef (n=349), chicken (n=325), pork (n=310), chevon (n=50), and meat products (n=100) were collected from different parts of Kerala, *viz*., Mannuthy, Thrissur, Angamali, Ernakulam, Calicut, and Wayanad. Raw meat samples were collected from markets and meat products such as chicken cutlet, chicken roll, beef cutlet, and beef roll were collected from retail outlets. 100 g of samples were collected aseptically in sterile polythene bags and transported to the laboratory under chilled condition. The samples were processed on the same day of collection.

### Enrichment and extraction of DNA

TSBYE (HiMedia, India) was selected as the universal enrichment broth for all the organisms under study based on the previous research [12].

A 25 g portion of each sample was aseptically transferred to 225 ml of TSBYE in a stomacher bag and homogenized in a stomacher (Smasher, AES, France) for 120 s and incubated at 37°C for 18 h. After enrichment, the samples were subjected to DNA extraction by boiling method [13] and multiplex PCR analysis. Culture-based detection for the four pathogens was also carried out in parallel.

### Multiplex PCR

The primers and the method used for screening samples were based on the multiplex PCR protocol developed by Latha *et al*. [14]. Multiplex PCR amplifications were conducted in a reaction mixture containing 2.0 μl PCR buffer (10×, Sigma), 2.0 μl MgCl_2_ (2.5 mM, Sigma), 1.0 μl dNTPs (2 mM each, Fermentas), 0.5 μl of each primer (10 µM, Sigma), 0.2 μl Taq Polymerase (5 U/μl, Sigma), and 2 μl of template DNA in a final volume of 25 μl. Amplification conditions were 2 min at 95°C, 35 cycles of 15 s at 95°C, 30 s at 60°C and 60 s at 72°C and a final extension of 10 min at 72°C. Reactions were carried out in BioRad T-100 Thermocycler.

### Microbiological analysis

Microbiological analysis was performed by the method demonstrated by APHA [15]. After enrichment, samples were plated onto polymyxin - acriflavine - lithium chloride - ceftazidime - aesculin - mannitol (PALCAM) agar (HiMedia, India) for *L. monocytogenes*, Yersinia selective agar (HiMedia, India) for *Y. enterocolitica*, Baird Parker agar (HiMedia, India) for *S. aureus* and Brilliant Green agar (HiMedia, India) for *S. enterica* Typhimurium. 10 presumptive colonies from each plate were selected for biochemical characterization. Biochemical identification of organisms under study was carried out as described previously [16]. Each sample was analyzed in triplicates.

## Results

### Prevalence of *L. monocytogenes*, *Y. enterocolitica*, *S. aureus*, and *S. enterica* Typhimurium in meat and meat products by multiplex PCR technique

All the samples were screened for the presence of *L. monocytogenes*, *Y. enterocolitica*, *S. aureus*, and *S. enterica* Typhimurium by the multiplex PCR protocol and results are given in Table 1.

**Table-1:** Prevalence of *L. monocytogenes, Y. enterocolitica, S. aureus,* and *S. enterica* Typhimurium in meat and meat products by multiplex PCR.

Samples	No. of samples	Prevalence in meat and meat products			

		*L. monocytogenes*	*Y. enterocolitica* (%)	*S. aureus* (%)	*S. enterica* Typhimurium
Beef	349	0	2 (0.57)	163 (46.70)	0
Chicken	325	0	0	143 (44)	0
Pork	310	0	2 (0.65)	149 (48.06)	0
Chevon	50	0	0	20 (40)	0
Meat products	100	0	0	23 (23)	0
Total	1134	0	4 (0.35)	498 (43.91)	0

*L. monocytogenes=Listeria monocytogenes, Y. enterocolitica=Yersinia enterocolitica, S. aureus=Staphylococcus aureus, S. enterica* Typhimurium=*Salmonella enterica* Typhimurium, PCR=Polymerase chain reaction

Out of 349 beef samples screened, *L. monocytogenes* and *S*. *enterica* Typhimurium were absent in all samples. *Y. enterocolitica* was present in two samples (0.57%). *S. aureus* could be detected from 163 (46.70%) samples. One sample was found to be positive for both *Y. enterocolitica* and *S. aureus*. *L. monocytogenes, Y. enterocolitica* and *S*. Typhimurium could not be isolated from any of the chicken samples screened. However, 143 samples were positive for *S. aureus* (44%).

Out of 310 pork samples, *L. monocytogenes* and *S*. Typhimurium were absent in all the samples, whereas *S. aureus* was present in 129 samples (41.61%). Two samples showed the presence of virulence gene; *ail* of *Y. enterocolitica* in the multiplex PCR. *nuc* gene of *S. aureus* was present in 20 samples of chevon and in 23 samples of meat products including five chicken cutlets, six beef cutlets, five chicken roll, and seven beef roll.

### Microbiological analysis of samples

In a total of 1134 samples collected, nine samples from beef, five samples each from chicken and pork, two samples from chevon, and one sample from meat products were positive for *Listeria* spp. and *L. monocytogenes* was identified in only one beef sample. Overall occurrence of *Listeria* spp. was 1.94%, and *L. monocytogenes* was 0.08%. *Yersinia* spp. was isolated from five beef and out of which one sample was identified as *Y. enterocolitica*. Two samples out of 325 chicken samples were positive for *Yersinia* spp. but none of the isolates were identified as *Y. enterocolitica*. Out of 310 pork samples screened, seven samples were positive for *Yersinia* spp. and two were confirmed as *Y. enterocolitica*. Chevon and meat products showed no positive colonies for *Yersinia* spp. and *Y. enterocolitica. S. aureus* showed high occurrence in all the type of samples screened. A total of 461 samples including 157 beef, 131 chicken, 137 pork, 16 chevon, and 20 meat products were positive for *S. aureus* by cultural examination. In a total of 349 beef samples collected, 106 samples were positive for *Salmonella* spp. Out of 325 samples of fresh chicken collected; *Salmonella* spp. was present in 98 samples. A total of 310 samples from pork were analyzed and 87 samples showed the presence of *Salmonella* spp. and out of 50 samples, 12 chevon samples showed the presence of *salmonella* spp. *Salmonella* colonies were also isolated from eight meat products. However, no colonies were identified as *S. enterica* Typhimurium.

## Discussion

With the rapid increase in global commerce, fast and cost effective detection methods for foodborne pathogens are important, particularly in the food industry, as they are able to detect the presence of pathogens in raw and processed foods immediately. Rapid methods are more time-efficient, labor-saving and able to reduce human errors; nevertheless, each of the rapid methods has its own advantages and limitations [17]. Multiplex PCR is a relatively efficient method that can simultaneously amplify template mixture and decrease the detection costs, conquering the weakness of single PCR detecting only one template once [18].

In this study, multiplex PCR was used as the rapid method to study the prevalence of bacterial pathogens in different kinds of meat and meat products. The assay was carried out according to the procedure developed by Latha *et al*. [12].

Various meats and meat products such as beef, chicken, pork, and chevon have been associated with *Listeria* contaminations [19]. In this study, overall occurrence of *Listeria* spp. was 1.94%, and *L. monocytogenes* was 0.08% by cultural methods. However, *hly A* gene of *L. monocytogenes* could not be identified in any of the samples by multiplex PCR. Similar result was also obtained by Chen *et al*. [20], and they reported that the other *Listeria* species like *L. innocua* which lacks *hly A* gene can also grow as *L. monocytogenes* in PALCAM plates. In a study by Zarei *et al*. [10], *L. monocytogenes* was detected in 2.8% of the beef and buffalo samples, and 4.3% of the lamb samples. According to the previous reports from Nigeria, *Listeria* species could not be isolated from Pork and goat samples [21]. No contamination with *L. monocytogenes* was reported in 200 beef carcasses, in Northern Ireland [22].

As shown in Table 1, only beef and pork samples are contaminated with *Y. enterocolitica*. According to Esnault *et al*. [23], 5.2% beef samples were contaminated with *Y. enterocolitica* while in this study, 0.57% samples were found positive. According to Liang *et al*. [24], consumption of pork is the main source for yersiniosis in human and healthy pigs are known to be the primary reservoir of *Y. enterocolitica*. Tan *et al*. [25] reported a high prevalence of *Y. enterocolitica* in raw pork meat (23.8%).

Similar to previous studies, we found a high prevalence of *S*. *aureus* in all types of meat tested. Occurrence of *S. aureus* in meat products was less compared to raw meat. High prevalence may be associated with human contamination during processing of meat. Abdalrahman *et al*. [26] reported that the prevalence of *S. aureus* in beef was 65.6% which is higher than the present study showed 46.7%. A study by Hanson *et al*. [27] reported a low prevalence of *S. aureus* in beef (6.9%), chicken (17.8%), and pork (18.2%).

According to Wang *et al*. [28] consumption of contaminated meat and meat products acts as the common sources of salmonellosis. In the study, prevalence of *Salmonella* species in beef was 30.37% which was higher than the report by Maradiga *et al*. [29] who has reported 10 percent prevalence in beef. High level of contamination of chicken meat with *Salmonella* was reported by Fearnley *et al*. [30]. Out of 325 retail chicken meat samples analyzed, *Salmonella* could be isolated from 30 samples. Similarly, Yang *et al*. [31] and Van *et al*. [32] obtained a prevalence of 54% and 53.3% of *Salmonella*, respectively, from chicken meat, which is high compared to this study. Freitas *et al*. [33] observed that none of the samples was positive for *S*. Typhimurium in their study which is in agreement with this study.

## Conclusion

Consumption of contaminated raw meat and meat products increases the chance of foodborne illnesses. To reduce the risk of contamination and infection, it is important to monitor the presence of pathogens in all stages of meat processing from farm to fork. Multiplex PCR assay used in this study can detect more than one pathogen simultaneously by amplifying more than one target gene in a single reaction, which can save time and labor cost.

## Authors’ Contributions

CL designed the study and initiated the research. CJA and VJA carried out collection of samples, standardization of procedure, draft and revision of manuscript. BS helped in interpretation of results. All authors read and approved the final manuscript.
